# Signaling in and out: long-noncoding RNAs in tumor hypoxia

**DOI:** 10.1186/s12929-020-00654-x

**Published:** 2020-05-05

**Authors:** Tse-Chun Kuo, Hsing-Jien Kung, Jing-Wen Shih

**Affiliations:** 1 Institute of Molecular and Genomic Medicine, National Health Research Institutes, Zhunan, Miaoli County, 35053 Taiwan, ROC; 2 Graduate Institute of Cancer Biology and Drug Discovery, College of Medical Science and Technology, Taipei Medical University, Taipei, 11031 Taiwan, ROC; 3 Ph.D. Program for Cancer Biology and Drug Discovery, College of Medical Science and Technology, Taipei Medical University, Taipei, 11031 Taiwan, ROC; 4 Department of Biochemistry and Molecular Medicine, Comprehensive Cancer Center, University of California at Davis, Sacramento, CA 95817 USA; 5 TMU Research Center of Cancer Translational Medicine, Taipei Medical University, Taipei, 110 Taiwan, ROC; 6 Ph.D. Program for Translational Medicine, College of Medical Science and Technology, Taipei Medical University, Taipei, 11031 Taiwan, ROC

**Keywords:** Tumor hypoxia, Long non-coding RNA, lncRNA, HIF-1α, Hypoxia-associated lncRNAs, HAL, Extracellular vesicles

## Abstract

Over the past few years, long non-coding RNAs (lncRNAs) are recognized as key regulators of gene expression at chromatin, transcriptional and posttranscriptional level with pivotal roles in various biological and pathological processes, including cancer. Hypoxia, a common feature of the tumor microenvironment, profoundly affects gene expression and is tightly associated with cancer progression. Upon tumor hypoxia, the central regulator HIF (hypoxia-inducible factor) is upregulated and orchestrates transcription reprogramming, contributing to aggressive phenotypes in numerous cancers. Not surprisingly, lncRNAs are also transcriptional targets of HIF and serve as effectors of hypoxia response. Indeed, the number of hypoxia-associated lncRNAs (HALs) identified has risen sharply, illustrating the expanding roles of lncRNAs in hypoxia signaling cascade and responses. Moreover, through extra-cellular vesicles, lncRNAs could transmit hypoxia responses between cancer cells and the associated microenvironment. Notably, the aberrantly expressed cellular or exosomal HALs can serve as potential prognostic markers and therapeutic targets. In this review, we provide an update of the current knowledge about the expression, involvement and potential clinical impact of lncRNAs in tumor hypoxia, with special focus on their unique molecular regulation of HIF cascade and hypoxia-induced malignant progression.

## Background

### Hypoxia-associated lncRNAs (HALs) emerging as newly driving factors in tumorigenesis

In rapidly growing solid tumors, hypoxia is a common, microenvironmental characteristics, caused by insufficient vascularization, and the high tumor metabolic demands [1]. Accumulating evidence has demonstrated that tumor hypoxia is involved in the initial oncogenic transformation, but is also tightly linked to aggressive cancer phenotypes, such as metastases, recurrences and resistance to therapy [2–4]. Upon hypoxia, to survive, cancer cells co-opt the fundamental adaptive responses to this stress through modulating the central mediator of hypoxic response, the hypoxia-inducible factor-1 (HIF-1) complex.

The HIF-1 complex is a heterodimeric assembly of bHLH-PAS (basic helix-loop-helix DNA binding proteins of the PER-ARNT-SIM family) transcriptional factors, comprised of a constitutively expressed, stable HIF-1β subunit and an oxygen-sensitive HIF-1α subunit that determines HIF-1 activity [5, 6]. In mammals, two HIF-1α homologs, HIF-2α and HIF-3α (also known as IPAS-1; inhibitory PAS (Per/Arnt/Sim) domain protein), have been identified. Similar to HIF-1α, HIF-2α is also sensitive to oxygen concentration and can interact with HIF-1β to form the HIF-2 heterodimeric complex. Due to the structural similarity in DNA binding and dimerization domains as well as the difference in their transactivation domains, HIF-1α and HIF-2α regulate both common as well as distinct sets of target genes. Meanwhile, HIF-3α, an isoform lacking the transactivation domain, has a dominant negative effect on HIF-dependent gene transcription [7, 8].

**Fig. 1:**
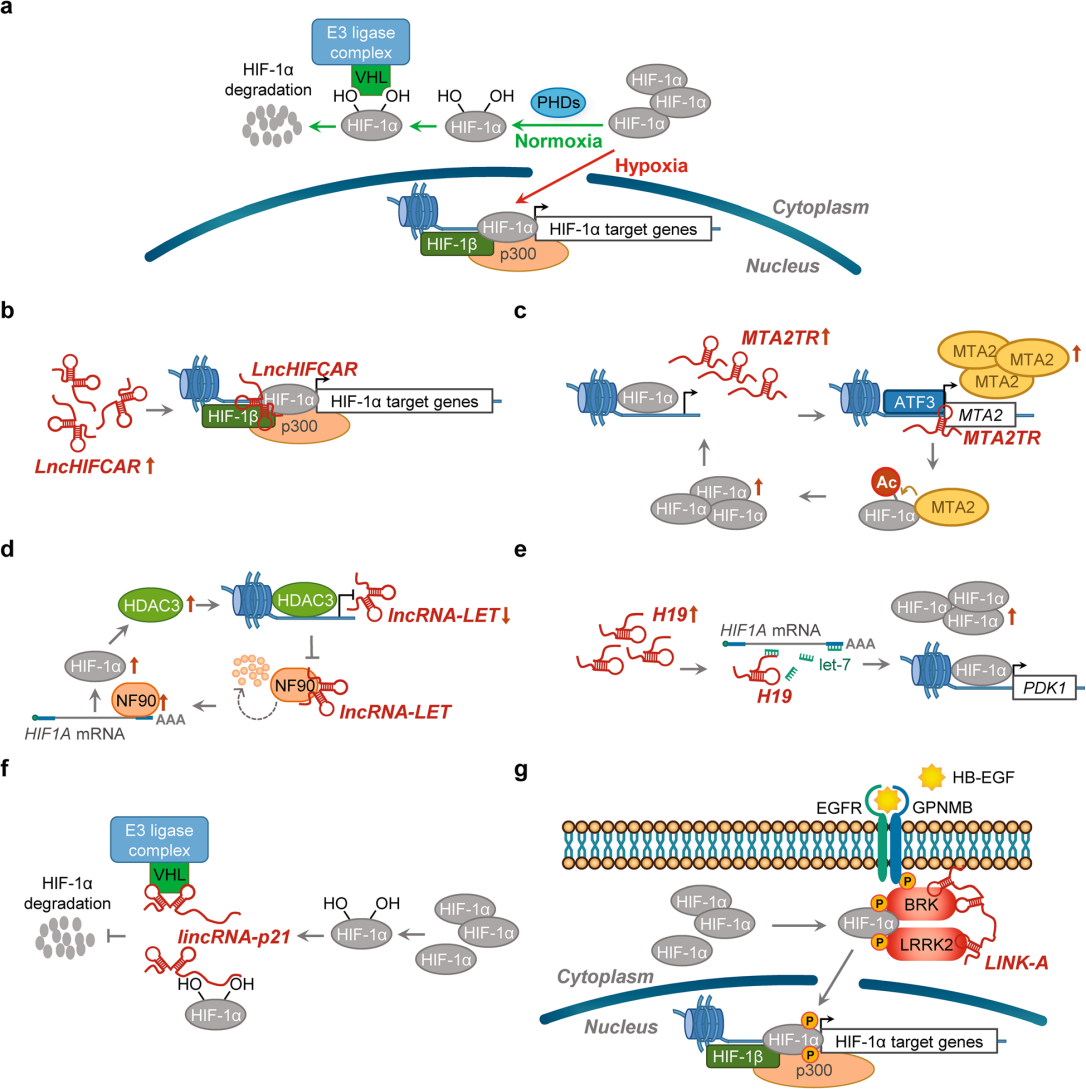
Regulations of HIF-1 activity by HALs. **a** Regulation of HIF-1. Under normoxia (green arrows), HIF-1α subunit is hydroxylated by PHDs (prolyl hydroxylase domain proteins). Hydroxylation residues within HIF-1α facilitates interaction of HIF-1α with the E3 ubiquitin ligase VHL protein, targeting HIF-1α for polyubiquitination and subsequent proteasome-dependent degradation. Upon hypoxia (red arrows), the PHDs and other prolyl hydroxylases are inhibited, leading to HIF-1α stabilization and translocation into nucleus. After dimerization with its transcriptional partner HIF-1β and recruitment of co-activators (e.g. CBP/p300), the HIF-1 heterodimer binds the HRE (hypoxia response element) of target genes to regulate transcription. **b** Transcriptional co-activator. Hypoxia-induced *LncHIFCAR* could directly interact with HIF-1α and facilitate the recruitment of HIF-1α and p300 cofactor to the target loci, thereby upregulating HIF-1 target genes. **c** Recruitment of transcription factor. HIF-1α-induced *LncRNA-MTA2TR* could recruit ATF3 to the promoter area of *MTA2*, thereby transcriptionally upregulating the expression of oncogenic MTA2. MTA2 can subsequently enhance HIF-1α protein accumulation via deacetylation, forming a feedback loop to amplify HIF-1 signaling. **d** mRNA stability control. The expression of *lncRNA-LET* is repressed through hypoxia-induced HDAC3, which reduces the histone H3 and H4 acetylation at the *LncRNA-LET* promoter. Decreased *lncRNA-LET* expression reduces the *lncRNA-LET*–mediated degradation of HIF-1α negative regulator, NF90, leading to HIF-1α accumulation. **e **ceRNA/miRNA sponge. Hypoxia-induced *H19* could upregulate HIF-1α expression by absorbing miRNA let-7 and nullifying let-7-mediated *HIF1A* mRNA suppression. **f **Molecular decoy. *lincRNA-p21* is able to disrupt the interaction between HIF-1α and its negative regulator VHL via separate binding to both HIF-1α and VHL, thereby blocking VHL-dependent HIF-1α degradation. **g **Complex scaffold. *LINK-A*-mediated recruitment and enzymatic activation of BRK and LRRK2 kinases could facilitate phosphorylation of HIF-1α at specific residues. These phosphorylation modifications prevent subsequent HIF-1α degradation and enhance the association between HIF-1α and cofactor p300, thereby upregulating HIF-1 target genes. See text for a more detailed discussion

In the presence of sufficient oxygen, HIF-1α subunits are post-translationally modified by a family of dioxygenases (prolyl hydroxylase domain-containing dioxygenases PHD1, 2 and 3, also known as EGLN1-3, Egl-9 family hypoxia inducible factor 1-3,). Upon hydroxylation, HIF-1α subunits are recognized by the E3 ubiquitin ligase, VHL (von Hippel-Lindau tumor suppressor protein), leading to the poly-ubiquitination and subsequent rapid degradation through the ubiquitin-proteasome pathway (Fig. 1a). Under hypoxic conditions, the PHD dioxygenase activity is inhibited, and the accumulated HIF-1α subunit translocates into the nucleus, dimerizing with HIF-1β and binding to the HREs (hypoxia response elements; the consensus 5′-(A/G)CGTG-3′ nucleotide sequence) within the promoter regions of HIF target genes to stimulate downstream transcriptional activation of multiple hypoxia responsive genes (Fig. 1a), eliciting a wide spectrum of cellular adaptations, such as decreased apoptosis, enhanced angiogenesis, proliferation, migration and invasion [1, 9–11]. In addition to protein coding genes, it has been widely acknowledged that the non-coding transcriptome is also responsive to hypoxia and play critical roles in the hypoxic response and HIF-1 associated cancer progression [12–16].

With recent advances in high-throughput sequencing, it is recognized that only a small fraction (< 2%) of the transcriptional output encodes proteins whereas the vast majority encode a variety of non-coding RNAs. Among these non-coding RNA species, long (> 200 bp) non-coding RNAs (lncRNAs) are a large class of regulatory transcripts [17], including lincRNAs (long intergenic RNAs), long intronic ncRNAs, pseudogenes, TCRs (transcribed ultra-conserved regions), asRNAs (antisense RNAs) and eRNAs (enhancer RNAs) [18]. According to the latest human genome annotation (GRch38, GENCODE release 33, January 2020; www.gencodegenes.org), 48,438 transcripts originating from 17,952 loci were identified as lncRNAs. Although less than 1% has been functionally annotated, growing evidence suggested the vital roles of these lncRNAs in regulation of gene expression at various stages, such as imprinting, transcription, RNA interference, RNA splicing, and translation control [19–23]. It is now believed that the distinctive RNA biochemical properties, such as base-pairing ability, dynamic expression and flexible structure, endow these lncRNAs with multi-functionality [24–28]. Collectively, it is now well appreciated that, through acting as signals, decoys, guides or scaffolds, lncRNA could act as a crucial player of biological regulation [23–25, 27, 29–33].

Over the last few years, a large number of dysregulated lncRNAs have been associated with numerous diseases, including cancer [34–37]. While a few cancer-associated lncRNAs have been well characterized [27, 38], the functions of most remain largely unknown. Dysregulation of many cancer-associated lncRNAs is linked to both clinicopathological features and survival outcomes of patients, suggesting that functional annotation of these lncRNAs will eventually identify new venues for early diagnosis and therapy of cancer [39]. Several studies have shown that the modulation of lncRNAs in response to hypoxia could play a regulatory role in HIF signaling cascade [14–16, 40, 41]. Here, we refer to these unique transcripts as “hypoxia-associated lncRNAs” (HALs). These RNA molecules are involved in multiple hypoxia-driven cancer progression pathways. In this review, we provide an updated summary of the tumor HALs, with a specific emphasis on the crosstalk between these lncRNA species and cellular hypoxia response (Table 1 and Additional file 1: Table S1). We address current models describing the functional involvement of these new players in cancer progression, highlighting their relevant clinical potential as cancer biomarkers or therapeutic targets. Our discussion is centered on tumor hypoxia. For the functional roles of lncRNAs in hypoxia-induced kidney/hepatic/myocardial injury and neuromuscular or cardiovascular diseases, interested readers are referred to a number of comprehensive reviews published in recent years [127–132].

**Table 1:** | HAL-mediated HIF signaling control and cancer progression

lncRNA	Status upon hypoxia	HIF involvement	Cancer Types	Clinical association	Functional Impact	Interactor	Target/Effect	Mechanistic Classification	Refs
*aHIF* *(HIF1A-AS2)*	Not further induced in nonpapillary disease, but can be induced in lymphocytes	N.D. (2 Putative HREs)	Renal carcinoma	• Up-regulated in non-papillary clear-cell renal carcinoma	N.D.	*HIF1A* mRNA	*HIF1A* mRNA stability	**mRNA stability control** (Binding of *HIF1A-AS2* to the *HIF1A* mRNA 3′-UTR could possibly expose AU-rich elements and thus increase the degradation of *HIF1A* mRNA)	[42, 43]
	Up-regulated	N.D.	Human umbilical vein endothelial cells (HUVECs)	• Up-regulated in HUVECs in hypoxia	HUVECs viability ↑ Migration ability ↑ Tube formation ↑	miR-153-3p	The expression of HIF-1α	**Sequestration of miRNAs** (Down-regulation of miR-153-3p-mediated repression of HIF-1α expression)	[44]
	Up-regulated	N.D.	Bladder cancer	• Upregulated in bladder cancer after cisplatin treatment	Cisplatin resistance ↑	N.D.	Promoting *HMGA1* expression	**Transcriptional regulation** (HIF1A-AS2 promoting the expression of *HMGA1*, which physically interacts with p53, p63, and p73, and therefore inhibits their transcriptional activity on *Bax*)	[45]
	Up-regulated	HIF-1α and/or HIF-2α dependent (2 HREs identified)	Mesenchymal Glioblastoma Stem-like Cells (M-GSCs)	• Upregulated in M-GSCs	Growth of M-GSCs ↑ Neurosphere-forming capacity of M-GSCs ↑ Glioblastoma tumor growth ↑	IGF2BP2 and DHX9	Maintenance of expression of *HMGA1*	**Complex scaffold** (The direct interaction among *HIF1A-AS2*, IGF2BP2 and DHX9 is needed for *HMGA1* expression)	[46, 47]
	Up-regulated	N.D.	Epithelial ovarian cancer (EOC)	• Up-regulated in EOC	Cell apoptosis ↓ Cell proliferation ↑ Tumorigenesis ↑ Tumor growth ↑	N.D.	N.D.	**Unclear mechanism** (May partially through the *aHIF*-mediated regulation of certain key mitochondrial apoptosis pathway-related genes, including Bcl-2, Bax, Caspase-7, and Caspase-9)	[48]
*AGAP2-AS1*	Up-regulated	N.D.	Hepatocellular carcinoma (HCC)	• Up-regulated in HCC • Correlated with adverse clinical features and poor prognosis of HCC	Cell proliferation ↑ Migration and invasion ↑ EMT progression ↑ Apoptosis ↓	miR-16-5p	The expression of ANXA11	**Sequestration of miRNAs** (Down-regulation of miR-16-5p-mediated repression of ANXA11)	[49]
*ANRIL (CDKN2B-AS1)*	Up-regulated	HIF-1α dependent (1 HRE identified)	Osteosarcoma	• Up-regulated in osteosarcoma	Hypoxic viability ↑ Hypoxia-induced Invasion ↑ Hypoxia-induced apoptosis ↓	N.D.	N.D.	**Unclear mechanism** (Possibly through epigenetic modification)	[50]
*BC005927*	Up-regulated	HIF-1α dependent (2 HREs identified)	Gastric cancer (GC)	• Up-regulated in GC • Correlated with higher tumor-node-metastasis stages and poorer prognoses	Metastasis ↑	N.D.	N.D.	**Transcriptional regulation** (The neighboring gene, *EPHB4*, a metastasis-related gene, is regulated by *BC005927*)	[51]
*BX111887 (ZEBTR)*	Up-regulated	HIF-1α dependent (1 HRE identified)	Pancreatic cancer (PC)	• Upregulated in PC • Correlated with late TNM stage, lymphatic invasion and distant metastasis	Proliferation ↑ Migration ↑ Invasion ↑	YB1	*ZEB1* promoter	**Transcriptional regulation** (*BX111* promotes *ZEB1* transcription by recruiting YB1 to *ZEB1* promoter)	[52]
*CASC9*	N.D.	N.D.	Nasopharyngeal carcinoma (NPC)	Up-regulated in NPC tissues	Glycolysis and tumorigenesis ↑ Cell growth ↑	HIF-1α	The stability of HIF-1α	**Protein Stability** (*CASC9* interacts with HIF-1α and enhances the stabilization of HIF-1α)	[53]
*CF129* *(lncRNA-CF129145.1)*	Down-regulated	Downregulated by binding of HIF-1α/HDAC1 complex to CF129 promoter	Pancreatic cancer (PC)	• Down-regulated in PC • Low CF129 expression predicted short overall survival	Invasion and metastasis ↓	p53 and E3 ligase MKRN1	*FOXC2* transcription	**Post-Translational modification** (*CF129* directly binds to p53 and E3 ligase MKRN1, inducing p53 protein ubiquitination and degradation, and thereby suppressing *FOXC2* transcription)	[54]
*CPS1-IT1*	Down-regulated (treatment of hypoxia mimetic, CoCl_2_)	N.D.	Colorectal cancer	Down-regulated in colorectal cancer	EMT and autophagy ↓	N.D.	N.D.	**Unclear mechanism** (May partially through suppressing expression levels of HIF-1α, LC3-I, LC3-II, Beclin-1 and EMT associated proteins under hypoxia)	[55]
*CRPAT4* *(RP11-225B17)*	Down-regulated	HIF-1α dependent, HIF-2α independent	Clear cell renal cell carcinoma (ccRCC)	• Up-regulated in ccRCC • Associated with poor overall survival and progression-free survival	Cell migration ↑ Proliferation ↑	N.D.	N.D.	**Unclear mechanism** (May partially through the *CRPAT4*-mediated regulation of migration-associated gene *AVL9* expression)	[56]
*DANCR*	N.D.	N.D.	Nasopharyngeal carcinoma (NPC)	• Up-regulated in NPC • Associated with poor prognosis	Metastasis ↑ Invasion ↑	NF90/NF45 complex	HIF-1α mRNA stability	**mRNA stability control** (*DANCR* could increase HIF-1α mRNA stability through interacting with the NF90/NF45 complex)	[57]
*DARS-AS1*	Up-regulated	HIF-1α dependent, But HIF-2α independent (2 HREs identified)	Myeloma	• Up-regulated in myeloma • Correlated with poor prognosis	Survival ↑ Tumorigenesis ↑	RBM39	RBM39 stability	**Post-Translational modification** (The interaction between *DARS-AS1* and RNA-binding protein 39 (RBM39) impedes the interaction between RBM39 and its E3 ubiquitin ligase RNF147, preventing RBM39 from degradation)	[58]
*EIF3J-AS1 (EIF3J-DT)*	Up-regulated	N.D.	Hepatocellular carcinoma (HCC)	• Up-regulated in HCC tissues • Correlated with tumor size, vascular invasion, tumor stage and poor prognosis	Cell proliferation ↑ Migration ↑ Invasion ↑	miR-122-5p	The expression of CTNND2	**Sequestration of miRNAs** (Down-regulation of miR-122-5p-mediated repression of CTNND2)	[59]
*ENST00000480739 (RPL13AP23)*	N.D.	N.D.	Pancreatic ductal adenocarcinoma (PDAC)	• Down-regulated in PDAC • Associated with tumor node metastasis (TNM) stage and lymph node metastasis • Independent risk factor for PDAC survival following surgery	Invasion ↓ *OS-9* mRNA & protein ↑	N.D.	Transcription of *OS-9* (Negative regulation of HIF-1α)	**Epigenetic and transcriptional regulation** (*ENST00000480739* induces *OS-9* expression at the transcriptional level, possibly through modifying the H3K27 acetylation level of *OS9* gene promoter)	[60]
*FALEC*	Up-regulated	HIF-1α inducible	Prostate cancer (PCa)	• Up-regulated in PCa • Independent prognostic factor	Cell proliferation ↑ Migration and invasion ↑	N.D.	N.D.	**Unclear mechanism** (May partially through the *FALEC*-mediated regulation of p21 and its downstream components expression)	[61]
*FAM201A*	N.D.	N.D.	Non-small cell lung cancer (NSCLC)	• Up-regulated in tissues obtained from NSCLC patients resistant to radiotherapy	Cell proliferation ↑ Apoptosis (under X-ray irradiation) ↓	miR-370	The expression of EGFR	**Sequestration of miRNAs** (Down-regulation of miR-370-mediated repression of EGFR)	[62]
*FEZF1-AS1*	N.D.	N.D.	Pancreatic cancer	• Upregulated in pancreatic cancer	Cell proliferation ↑ Invasion ↑	miR-142 and miR-133a	The expression of HIF-1α and EGFR	**Sequestration of miRNAs** (Down-regulation of miR-142- and miR-133a-mediated repression of HIF-1α and EGFR expression)	[63]
*GAPLINC*	Up-regulated	HIF-1α (2 HREs identified) (2 HREs)	Gastric cancer	• Upregulated in GC • High expression of *GAPLINC* correlates with poorer survival • GAPLINC correlates with CD44 activation	Proliferation ↑ Apoptosis ↓ Invasion ↑ Migration ↑	miR-211-3p	The expression of CD44	**Sequestration of miRNAs** (Down-regulation of miR-211-3p-mediated repression of CD44)	[64, 65]
*H19*	Up-regulated	N.D.	Breast cancer stem cells (BCSCs)	• H19 expression strongly correlates with PDK1 in primary breast carcinomas	Glycolysis ↑ BCSC maintenance ↑	let-7	The expression of HIF-1α	**Sequestration of miRNAs** (Down-regulation of let-7-mediated repression of HIF-1α expression)	[66]
	Up-regulated	N.D.	Multiple Myeloma (MM)	N.D.	The expression of the hypoxia induced genes ↑ Adhesion on stromal cells ↑	N.D.	N.D.	**HIF-1α nuclear translocation** (*H19* is required for HIF-1α nuclear translocation and the expression of the hypoxia-induced genes, such as CXCR4 and Snail)	[67]
	Up-regulated	HIF-1α dependent (3 HREs identified)	Glioblastoma (GBM)	• Up-regulated in GBM • Correlated with poor prognosis • The HIF-1α levels were positively correlated with H19 levels in GBM specimens	Migration and invasion ↑ Tumor growth ↑ EMT ↑	miR-181d	The expression of β-catenin	**Sequestration of miRNAs** (Down-regulation of miR-181d-mediated repression of β-catenin expression)	[68–71]
	Up-regulated	N.D.	Prostate Cancer	• Upregulated by estrogen or hypoxia • Reduced upon combined treatment	Cell motility ↓ Invasion ↓	N.D.	Repression of beta3 and beta4 Integrins	**Unclear mechanism** (Combined Estrogen and Hypoxia treatment could cause H19 down-regulation, followed by up-regulation of both β3 and β4 Integrins and E-cadherin)	[72]
	Up-regulated	N.D.	Breast cancer, Non-small cell lung carcinoma (NSCLC)	• Up-regulated in NSCLC with chronic obstructive pulmonary disease (COPD) • Up-regulated in all common metastatic sites tested	Migration and invasion ↑ Tumor growth ↑ EMT ↑	N.D.	Up-regulation of miR-675-5p	**Unclear mechanism** (*H19* could induce upregulation of miR-675-5p, whereas P53 is a target gene of miR-675-5p and P53 downstream target genes involved in EMT, survival and tumorigenesis are thereby repressed)	[73, 74]
*HAS2-AS1*	Up-regulated	HIF-1α dependent (1 HRE identified)	Oral squamous cell carcinoma (OSCC)	• Up-regulated in OSCC	EMT ↑	N.D.	N.D.	**Unclear mechanism** (*HAS2-AS1*-mediated hypoxia-induced EMT is dependent on cell-adhesion molecule CD44 and RHAMM)	[75]
*HIF2PUT*	N.D.	N.D.	Osteosarcoma	• Expression of *HIF2PUT* is correlated with *HIF2A* mRNA	Cell proliferation and migration ↓ Expression of CSC marker CD133 ↓ Sphere-forming ability ↓	N.D.	Transcription of *HIF2A*	**Transcriptional regulation** (HIF-2α was positively regulated by lncRNA *HIF2PUT*)	[76]
	N.D,	N.D.	Osteosarcoma cancer stem cell	• Down-regulated in osteosarcoma cell lines • A strong positive correlation between relative HIF2PUT and HIF-2α level in osteosarcoma cancer tissues	Proliferation ↓ Migration and invasion ↓ Sphere-formation ↓	N.D.	N.D.	**Unclear mechanism** (May partly through *HIF2PUT*-mediated regulation of HIF-2 expression)	[77]
*HINCUT-1 (uc.475)*	Up-regulated	HIF-1α dependent (3 HREs identified)	Colon and breast cancer cell lines	N.D.	Hypoxic cell proliferation ↑	N.D.	N.D.	**Transcriptional regulation** (*HINCUT-1* is required for the expression of *OGT* mRNA expression and global O-GlcNAcylation of proteins)	[78]
*HOTAIR*	N.D.	N.D.	Renal cell carcinoma	• Upregulated and correlated with tumor progression	RCC proliferation ↑ Migration and EMT ↑ Apoptosis ↓	miR-217	The expression of HIF-1α/AXL	**Sequestration of miRNAs** (Down-regulation of miR-217-mediated repression of HIF-1α/AXL expression)	[79]
	Up-regulated	HIF-1α dependent (1 HRE identified)	Non-small cell lung carcinoma (NSCLC)	• High level of *HOTAIR* is associated with poor clinical outcome in multiple cancers	Cell proliferation under hypoxia ↑ Invasion & migration under hypoxia ↑ Apoptosis under hypoxia ↓	N.D.	N.D.	**Unclear mechanism** (Possibly through *HOTAOR*-mediated epigenetic modification)	[80, 81]
*HOTTIP*	Up-regulated	HIF-1α dependent	Glioma	• Up-regulated in glioma • Associated with metastasis and poor patient survival	EMT ↑ Invasion ↑ Migration ↑	miR-101	The expression of *ZEB1*	**Sequestration of miRNAs** (Down-regulation of miR-101-mediated repression of *ZEB1*)	[82]
*IDH1-AS1*	N.D.	N.D. (c-Myc-mediated repression)	Multiple cell lines (HeLa, HCT116, H1299, P493 and 293 T)	N.D.	Glycolysis ↓	IDH1	IDH1 dimerization	**Protein Dimerization** (*IDH1-AS1* interacts with IDH1 and promotes Its Homo-dimerization)	[83]
*LINC01436*	Up-regulated	N.D.	Non-small cell lung cancer (NSCLC)	• Up-regulated in NSCLC • Associated with poor overall survival	Cell growth ↑ Migration and invasion ↑	miR-30a-3p	The expression of EPAS1	**Sequestration of miRNAs** (Down-regulation of miR-30a-3p-mediated repression of EPAS1)	[84]
*lincRNA-p21 (TP53COR1)*	Up-regulated	HIF-1α dependent & preference (2 HREs identified)	Cervical, lung and breast cancer cell lines	N.D.	Hypoxic glycolysis ↑ Tumor growth ↑	HIF-1α and VHL	The disruption of the VHL-HIF-1α interaction	**Protein-Protein Interaction Decoy** (Stabilization of HIF-1α by disrupting the VHL-HIF-1α Interaction)	[85]
	Up-regulated	N.D.	Hepatoma, glioma	N.D.	Apoptosis ↓ Cell proliferation and motility ↑ Autophagy ↑	N.D.	N.D.	**Unclear mechanism** (*LincRNA-p21* could promote autophagy of hypoxic tumor cells by up-regulating HIF-1α protein levels and suppressing Akt/mTOR/P70S6K signaling pathways)	[86]
*linc-ROR*	Up-regulated	N.D.	Hepatocellular cancer	Up-regulated in malignant liver cancer cells	Cell viability during hypoxia ↑ Tumor growth ↑	miR-145	The expression of *p70S6K1* (*RPS6KB1*)	**Sequestration of miRNAs** (Down-regulation of miR145-mediated repression of p70S6K1 expression)	[87]
*LINK-A* *(LINC01139)*	N.D.	N.D.	Triple-negative breast cancer	• Upregulated in TNBC • High levels of *LINK-A* correlated with unfavorable recurrence-free survival for breast cancer patients	Glycolysis ↑ Tumor growth ↑	BRK and LRRK2 kinase	HIF-1α phosphorylation	**Complex scaffold** (*LINK-A* facilitates the recruitment of BRK and LRRK2 kinase activation, thereby causing HIF-1α stabilization, HIF-1α/p300 interaction, and activation of HIF-1α transcriptional programs under normoxic conditions)	[88]
*LncHIFCAR (MIR31HG)*	Up-regulated	HIF-1α dependent	Oral cancer	• Up-regulated in oral cancer • High levels of *LncHIFCAR* predicted worse overall survival and recurrence-free survival	Hypoxic glycolysis ↑ Tumor metastasis ↑ Invasion and migration ↑ Hypoxic cell proliferation ↑ Sphere-forming ability ↑	HIF-1α	Activation of HIF-1 signaling	**Transcriptional regulation** (*LncHIFCAR* acts as HIF-1α coactivator)	[89]
*lncRNA-AK058003*	Up-regulated	N.D.	Gastric cancer	Up-regulated in GC	Invasion & migration ↑ Metastasis ↑	N.D.	N.D.	**Epigenetic regulation** (*AK058003* expression is positively correlated with *SNCG* expression and *SNCG* promoter demethylation)	[90]
*lncRNA-EFNA3*	Up-regulated	HIF-1α dependent (1 HRE identified)	Breast cancer	A strong correlation between high *EFNA3* expression and shorter metastasis-free survival in breast cancer patients	Cell extravasation ↑ Metastatic dissemination ↑	miR-210	The expression of EFNA3	**Sequestration of miRNAs** (Down-regulation of miR-210-mediated repression of EFNA3)	[91]
*lncRNA-HAL* *(lnc-METTL16-2)*	Up-regulated	HIF-1α dependent (3 putative HREs found)	Breast cancer	Up-regulated in triple negative breast cancer	Migration ↑ Cancer stem cell phenotype ↑ Mammospheres ↑ Clonogenic growth ↑	Histones and hnRNPs.	N.D.	**Unclear mechanism** (The binding of *lncRNA-HAL* to histones and hnRNPs may suggest a participation at the chromatin level and transcriptional regulation)	[92]
*lncRNA-LET (NPTN-IT1)*	Down-regulated	HIF-1α dependent (Indirect: Histone deacetylation)	Lung squamous-cell cancer (LSCC), hepatocellular carcinoma (HCC) and colorectal cancer (CRC)	• Down-regulated in in LSCC, HCC and CRC • Correlated with hypoxia, histone acetylation disorder and metastasis in HCC	Metastasis ↓ Invasion ↓	NF90 (RNA-binding protein)	*HIF1A* mRNA stability	**mRNA stability control** (The association between *lncRNA-LET* and NF90 protein enhanced the degradation of NF90, thereby decreasing *HIF1A* mRNA)	[93]
*lncRNA-SARCC* *(lnc-P2RY1-1)*	VHL-dependent	HIF-2α dependent (1 HRE identified)	Renal cell carcinoma	Differentially regulated by hypoxia in a von Hippel-Lindau (VHL)-dependent manner in RCC clinical specimens	Hypoxic cell cycle progression (VHL-restored RCC cells) ↑ Hypoxic cell cycle progression (VHL-mutant RCC cells) ↓	AR (androgen receptor)	AR ubiquitination and degradation	**Post-Translational modification** (*lncRNA-SARCC* could promote AR degradation via ubiquitin-mediated proteolysis to suppress AR/HIF-2α/C-MYC signals)	[94]
*lncTCF7 (WSPAR)*	Up-regulated	N.D.	Glioma	• Up-regulated in glioma • Associated with WHO grade and tumor size	Cell migration ↑ Proliferation ↑ Tumorigenicity ↑	N.D.	N.D.	**Unclear mechanism** (*LncTCF7* could promote the migration and proliferation of glioma cell partially through activating the Wnt signalling pathway)	[95]
*MALAT1*	Up-regulated	HIF-2α dependent & preference (1HRE)	Hepatocellular carcinoma	N.D.	Cell growth ↑ Glycolysis ↑ Migration & invasion ↑ Vasculature formation ↑ Metastasis ↑	N.D.	N.D.	**Post-Translational modification** (*MALAT1* decreases hydroxylation of HIF-1α/HIF-2α, possibly through disassociation of the VHL protein from HIF-1α/HIF-2α)	[96, 97]
	Up-regulated	N.D.	Lung adenocarcinoma	N.D.	Proliferation ↑ Migration ↑ Invasion ↑	PTB-associated splicing factor (PSF)	*GAGE6* promoter	**Transcriptional regulation** (The physical interaction of *MALAT1* and PSF released the binding of PSF to *GAGE6* promoter)	[98, 99]
	Up-regulated	N.D.	Hepatocellular carcinoma	N.D.	Proliferation ↑ Migration and invasion ↑ Apoptosis ↓	miR-200a	N.D.	**Sequestration of miRNAs** (Down-regulation of miR-200a)	[100]
*MEG3*	Up-regulated	N.D.	Pheochromocytoma	N.D.	Hypoxia-induced PC12 cell injury ↑	Methylation proteins (DNMT3a, DNMT3b, and MBD1)	*TIMP2* promoter methylation	**Epigenetic regulation** (*MEG3* recruited methylation proteins DNMT3a, DNMT3b, and MBD1 and accelerated *TIMP2* promoter methylation, which in turn inhibited its expression)	[101]
*MTA2TR*	Up-regulated	HIF-1α dependent (1 HRE identified)	Pancreatic cancer (PC)	Upregulated in PC tissues	Cell proliferation ↑ Invasion ↑	Activating transcription factor 3 (ATF3)	The expression of MTA2 (MTA2 stabilizes the HIF-1α via deacetylation)	**Transcriptional regulation** (*MTA2TR* transcriptionally upregulates MTA2 expression by recruiting ATF3 to the promoter area of *MTA2*)	[102]
*NEAT1*	Up-regulated	HIF-2α dependent	Non-small cell lung cancer (NSCLC)	• Up-regulated in NSCLC • Associated with TNM stage and metastasis	Cell proliferation ↑ Migration and invasion ↑	miR-101-3p	SOX9/Wnt/β-catenin signaling pathway	**Sequestration of miRNAs** (Down-regulation of miR-101-3p-mediated repression of SOX9/Wnt/β-catenin signaling pathway)	[103]
	Up-regulated	HIF-2α dependent & preference	Breast cancer	High expression of *NEAT1* is associated with poor survival of breast cancer patients	Proliferation ↑ Apoptosis ↓ Clonogenic survival ↑ Paraspeckle formation ↑	N.D.	N.D.	**Complex scaffold** (Induces paraspeckle formation, thereby enhancing cancer cell survival in hypoxia)	[12, 104–106]
*NDRG-OT1* *(lnc-NDRG1-1)*	Up-regulated	N.D.	Breast cancer	• N.D.	N.D.	NDRG1	NDRG1 degradation	**Post-Translational modification** (*NDRG-OT1* could promote NDRG1 degradation via ubiquitin-mediated proteolysis)	[107]
*NORAD*	Up-regulated	N.D.	Pancreatic cancer (PC)	• Upregulated in PC • Correlated with shorter overall survival	Migration ↑ Invasion ↑ EMT ↑ Metastasis ↑	miR-125a-3p	The expression of RhoA	**Sequestration of miRNAs** (Down-regulation of miR-125a-3p-mediated repression of RhoA)	[108]
*NUTF2P3-001* *(NUTF2P3)*	Up-regulated	HIF-1α dependent (1 HRE identified)	Pancreatic cancer	• Upregulated in pancreatic cancer • A positive correlation between NUTF2P3 and KRAS • Associated with tumor stage and prognosis	Cell viability, proliferation ↑ Invasion ↑ KRAS expression ↑ Metastasis ↑	miR-3923	The expression of KRAS	**Sequestration of miRNAs** (Down-regulation of miR-3923-mediated repression of KRAS)	[109]
*PCGEM1*	Up-regulated	N.D.	Gastric cancer (GC)	Up-regulated in GC	Invasion and metastasis ↑	N.D.	N.D.	**Unclear mechanism** (Partially through regulating SNAI1, a key transcription factor of EMT)	[110]
*PVT1*	N.D.	N.D.	Nasopharyngeal carcinoma (NPC)	• Up-regulated in NPC • Up-regulation is associated with a poor prognosis in NPC patients	NPC cell proliferation ↑ Colony formation ↑ In vivo tumorigenesis ↑	KAT2A (chromatin modification factor)	Transcription of *NF90* (RNA-binding protein)	**Epigenetic regulation** (*PVT1* serves as a scaffold for KAT2A, which mediates H3K9 acetylation, recruiting the nuclear receptor binding protein TIF1β to activate *NF90* transcription, thereby increasing HIF-1α mRNA stability)	[111]
	N.D.	N.D.	Hepatocellular carcinoma (HCC)	Up-regulated in HCC tissues and cell lines	Cell proliferation ↑ Migration ↑ Invasion and iron uptake ↑ Apoptosis ↓	miR-150	The expression of HIG2 (Hypoxia-inducible protein 2)	**Sequestration of miRNAs** (Down-regulation of miR-150-mediated repression of HIG2)	[112]
	N.D.	N.D.	Gastric cancer	• Upregulated in GC tissues and cell lines • High expression levels correlated with advanced tumor stage and lymph node metastasis	GC cell proliferation ↑ GC cell invasion ↑	miR-186	The expression of HIF-1α	**Sequestration of miRNAs** (Down-regulation of miR-186-mediated repression of HIF-1α expression)	[113]
	Up-regulated	N.D.	Non-small cell lung cancer (NSCLC)	• Up-regulated in HIF-1α high group compared with HIF-1α low group • Negatively correlated with miR-199a-5p expression in NSCLC tissues	Cell proliferation ↑	miR-199a-5p	The expression of HIF-1α	**Sequestration of miRNAs** (Down-regulation of miR-199a-5p-mediated repression of HIF-1α expression)	[114]
	Up-regulated treatment of hypoxia mimetic CoCl_2_)	N.D.	Cervical Cancer	• Up-regulated in Cervical cancer • Correlates with poorer overall survival	Cell proliferation ↑ Migration and invasion ↑ Apoptosis ↓ Cisplatin resistance ↑	N.D.	N.D.	**Unclear mechanism** (Possible involvement of the interaction with nucleolin)	[115]
*RERT-lncRNA* *(RAB4B-EGLN2)*	N.D.	N.D.	Hepatocellular carcinoma (HCC)	The expression levels of *RERT-lncRNA* and *EGLN2* were significantly correlated in HCC	*EGLN2* expression ↑	N.D.	N.D.	**Transcriptional regulation** (*RERT-lncRNA* induces *EGLN2/PHD1* expression at the transcriptional level)	[116]
*UBE2CP3*	N.D.	N.D.	Hepatocellular carcinoma (HCC)	• Up-regulated in HCC, especially in high EV (endothelial vessel) density tissues • UBE2CP3 expression combined with EV density is associated with HCC patient prognosis	Proliferation ↑ Migration ↑ Tube formation ↑	N.D.	N.D.	**Unclear mechanism** (May partially through UBE2CP3-induced increase in the secretion of VEGFA into the supernatant via activation of the ERK/HIF-1α signaling pathway)	[117]
*UCA1*	Up-regulated	HIF-1α-dependent	Estrogen receptor (ER)-positive breast cancer	N.D.	Tamoxifen resistance ↑	miR-18a	The expression of HIF-1α	**Sequestration of miRNAs** (Down-regulation of miR-18a-mediated repression of HIF-1α expression)	[118]
	Up-regulated	N.D.	Hypoxia-resistant gastric cancer (HRGC)	Upregulated in HRGC cells	Migration ↑	miR-7-5p	The expression of EGFR	**Sequestration of miRNAs** (Down-regulation of miR-7-5p-mediated repression of EGFR)	[119]
	Up-regulated	N.D.	Acute myeloid leukemia (AML)	Upregulated following ADR (adriamycin)-based chemotherapy	Cytotoxic effect of ADR ↓ HIF-1α-dependent glycolysis ↑	miR-125a	The expression of HK2	**Sequestration of miRNAs** (Down-regulation of miR-125a-mediated repression of HK2)	[120]
	Up-regulated	HIF-1α dependent (2 HREs)	Bladder cancer	• Upregulated in bladder cancer • *UCA1* expression associated with the clinical stage and histologic grade of bladder cancer	Cell proliferation under hypoxia ↑ Invasion & migration under hypoxia ↑ Apoptosis under hypoxia ↓	N.D.	N.D.	**Unclear mechanism** (*UCA1* could modulate the expression of several genes involved in tumorigenic potential, drug resistance and embryonic development)	[121, 122]
	Up-regulated	HIF-1α dependent (1 HRE identified)	Osteosarcoma	N.D.	Cell growth ↑	N.D.	N.D.	**Unclear mechanism** (May partially through inactivating the PTEN/AKT signaling pathway)	[123]
*WT1-AS*	Up-regulated	HIF-1 dependent (DNA demethylation of the CpG island)	Myeloid Leukemia	• Upregulated in Wilms’ tumors • Aberrant WT1-AS splicing often found in acute myeloid leukemia	N.D.	N.D.	N.D.	**Epigenetic regulation** (*WT1-AS* mediates hypoxia-induced *WT-1* mRNA upregulation through modulating histone methylation)	[124, 125]
*ZEB2-AS1*	Up-regulated	HIF-1α dependent	Gastric cancer (GC)	• Upregulated in GC • Correlated with poor differentiation, lymph node metastasis and distant metastasis	Cell proliferation and growth ↑ Invasion ↑ In vivo tumor growth ↑	miR-143-5p	The expression of HIF-1α	**Sequestration of miRNAs** (Down-regulation of miR-143-5p-mediated repression of HIF-1α expression)	[126]

Abbreviation: *CRC* colorectal cancer, *CSC* cancer stem cell, *EMT* epithelial–mesenchymal transition, *GC* Gastric cancer, *HCC* hepatocellular cancer, *HRE* hypoxia response element, *HUVECs* human umbilical vein endothelial cells, *ICC* Immunocytochemistry, *LC* lung cancer, *M-GSCs* Mesenchymal glioblastoma multiforme stem-like cells, *N.D.* Not determined, *NSCLC* non-small cell lung carcinoma, *OSCC* Oral squamous cell carcinoma, *PDAC* pancreatic ductal adenocarcinoma, *RCC* Renal Cell Carcinoma, *RNP* ribonucleic protein, *TNM* tumor, node, metastasis, *VHL* von Hippel-Lindau protein, *WHO* World Health Organization

## Review

### LncRNAs as emerging driving forces in cancer progression upon tumor hypoxia

Given the pivotal roles of lncRNA in hypoxia-associated tumorigenesis pathways, multiple approaches have been applied in the identification of hypoxia-regulated lncRNAs [87, 90]. A comprehensive analysis coupling RNA-seq with ChIP-seq [12] revealed the extensive involvement of HIF-1α and HIF-2α in the transcriptional regulation of lncRNAs upon hypoxia. In recent years, the rapid expansion of research on lncRNAs has provided additional insights into those associated with cellular hypoxia response. Table 1 presents an updated list of these hypoxia-associated lncRNAs (HALs). Upon hypoxia, most HALs are up-regulated. HIF could directly promote the expression of these hypoxia-inducible lncRNAs through binding to the HREs (hypoxia response elements) located in their promoter (Table 1) [41]. *lncRNA-LET* [93], *CF129* [54] and *CRPAT4* [56] are among the few which are down-regulated in hypoxic conditions. Notably, *lncRNA-SARCC* is able to respond to hypoxic stress differentially in a VHL-dependent manner [94].

Most of the HALs identified have impacts on cancer progression, although the mechanistic details are not all clear. Table 1 shows an overview of the tumor HALs. We summarize in the table, their potential molecular target related to hypoxic responses as well as their reported functions and signaling pathways. These HALs may also have hypoxia-independent functions. For the sake of conciseness, those targets are not included in the table. In addition, some of these lncRNAs can be captured by exosomes and transmitted to tumor microenvironment to exert their functions and further propagate the hypoxic responses (Table 2). Notably, several HALs, such as *UCA1*, *PVT1*, *H19* and *MALAT1*, might adapt more than one action mode in different cancer types. In the discussion below, we highlight the selected few HALs to illustrate their mechanisms of actions.

**Table 2:** | HALs identified extracellularly

LncRNA	Extracellular space identified	Cell to Cell Transfer	Functional Impact	Mechanism	Ref
*aHIF* (*HIF1A-AS2*)	Serum (aHIF level in serum correlates with its expression in matched ectopic endometria)	Endometriotic cyst stromal cells (ECSCs)-derived exosomes to human umbilical vein endothelial cells (HUVECs)	Elicits proangiogenic behavior in HUVECs, thus facilitating endometriosis angiogenesis.	Activates VEGF-A, VEGF-D, and b-FGF in HUVECs	[133]
*CCAT2*	Exosomes secreted from cultured glioma cells	U87-MG glioma cells to HUVECs	Promotes HUVEC angiogenesis and inhibits apoptosis induced by hypoxia	Promotes VEGF-A, TGF-β and Bcl2 expression. Inhibits BAX and caspase 3 expression	[134]
*HISLA* (*LINC01146*)	Extracellular vesicles secreted by tumor associated fibroblasts (TAMs)	TAMs to breast cancer cells	Enhances aerobic glycolysis and apoptotic resistance of cancer cells	Stabilizes HIF-1α	[135]
*PVT1*	Exosomes secreted from cultured colon cancer cells. Cancer cells with more aggressive phenotypes have more extracellular *PVT1*	Not determined	Promotes cell proliferation and inhibits apoptosis.		[136]
*linc-ROR*	Exosomes secreted from cultured hepatocellular carcinoma cells	HCC cancer cells to cancer cells	Promotes cell survival of recipient cells	Through a miR-145–HIF-1α signaling module to increase HIF-1α expression	[87]
*UCA1*	Exosomes secreted from cultured bladder cancer cells & serum	Bladder cancer 5637 cells with high expression of UCA1 to bladder cancer UMUC2 cells with low expression of UCA1	Promotes cell proliferation, migration and invasion of recipient cells Promotes xenograft growth	Through regulating the expression of genes involved in EMT (E-cad, MMP9, vimentin)	[137]

#### HAL-mediated epigenetic and transcriptional regulation

A large number of lncRNAs are localized in the nucleus, participating in various biological processes, including chromatin organization, nuclear structure, transcriptional and post-transcriptional regulation of gene expression. With regard to chromatin organization, the pangenomic investigations of RNA–protein interactions have shown that two hypoxia-inducible, oncogenic antisense RNAs *ANRIL* (also known as CDKN2B antisense RNA 1) and *HOTAIR* (HOX transcript antisense RNA) [50, 80] could interact with different histone-modifying complexes, and have thus been proposed to impact the chromatin modification and transcriptional state [138]. However, whether these two antisense RNAs are involved in modulating gene expression in response to hypoxia via epigenetic modification or chromatin re-organization remains to be characterized. In addition, *WT1-AS* could mediate hypoxia-induced upregulation of oncogenic transcription factor WT-1 *in cis* through modulating histone H3K4 and H3K9 methylation around the transcription start site of *WT1* mRNA, contributing to acute myeloid leukemia (AML) progression [124]. Similarly, in gastric cancer, *lncRNA-AK058003*, which could be profoundly induced by hypoxia, resides upstream of *SNCG* (synuclein gamma, a synuclein family member, promotes migration, invasion and metastasis) and enhances *SNCG* expression *in cis* through demethylation of *SNCG* promoter CpG islands, thereby driving hypoxia-induced metastasis [90]. In the context of nasopharyngeal carcinoma (NPC), up-regulated *PVT1* could serve as a scaffold for a transcriptional activator, the histone acetyltransferase KAT2A, to activate transcription of *NF90*. NF90, a RNA-binding protein, has been reported to stabilize many target mRNAs, including *HIF1A* mRNA. Indeed, the upregulated NF90 increased *HIF1A* mRNA stability and promoted malignant transformation of NPC cells [111]. In addition, in hypoxia-injured pheochromocytoma cells, up-regulated *MEG3* (maternally expressed gene 3) could recruit methylation proteins DNMT3a, DNMT3b and MBD1 to facilitate *TIMP2* promoter methylation, which in turn inhibited the expression of this cell cycle arrest inducer TIMP2. Moreover, a HIF-1α negative regulator, OS-9, is reported to facilitate HIF-1α hydroxylation and subsequent proteasomal degradation through tethering the interaction between HIF-1α and prolyl hydroxylases (PHDs) [139]. Interestingly, in pancreatic ductal adenocarcinoma (PDAC), another lncRNA *ENST00000480739* could inhibit HIF-1α by up-regulating *OS9* (osteosarcoma amplified-9) expression through enhancing the acetylation of H3K27 within *OS9* gene promoter [60]. Of note, in PDAC, the level of *ENST00000480739* is markedly downregulated, and negatively correlated with lymph node metastasis, in agreement with its negative regulatory role in HIF-1 signaling [60]. As *ENST00000480739* resides upstream of the *OS9* promoter region, this lncRNA also act *in cis* to induce *OS9* transcription.

Apart from chromatin structure remodeling, a series of HALs could modulate transcription and thereby fine-tune the HIF network. For instance, lncRNA *HIF2PUT* (HIF-2α promoter upstream transcript), *RERT-lncRNA* and hypoxia-inducible *BC005927* are all found to act *in cis* to up-regulate neighboring protein-coding genes *HIF2A* (encodes HIF-2α), *EGLN2* (encodes prolyl hydroxylase PHD1) and *EPHB4* (encodes Ephrin type-B receptor 4, a metastasis-related gene), at the transcriptional level, respectively [51, 76, 116].

Moreover, HALs could directly act on specific transcription factors through physical interactions to modulate their transactivation activities. We recently identified a hypoxia-inducible lncRNA *LncHIFCAR* (long noncoding HIF-1α co-activating RNA, also known as *MIR31HG*) acting as a HIF-1α co-activator via direct interaction with HIF-1α, thereby enhancing the binding of HIF-1α and cofactor p300 to the target loci (Fig. 1b). As the abundance of the HIF complex increases, the hypoxia-induced HIF-1 signaling cascade is augmented to further promote subsequent cancer progression [89]. Meanwhile, in pancreatic cancer, HIF-1α-induced lncRNA*-MTA2TR* (MTA2 transcriptional regulator RNA) transcriptionally up-regulates the expression of oncogenic MTA2 (metastasis associated protein 2) by recruiting ATF3 (activating transcription factor 3) to the promoter area of *MTA2* [102]. Subsequently, MTA2 can enhance the accumulation of HIF-1α protein via MTA2-mediated HIF-1α deacetylation and stabilization, which further activates HIF-1α transcriptional activity, forming feedback loops to augment HIF-1 signaling [102] (Fig. 1c). In addition, through binding to PSF (PTB-associated splicing factor), hypoxia-induced lncRNA *MALAT1* released PSF from its downstream proto-oncogene *GAGE6* (proto-oncogene G antigen 6) and activated its transcription, thereby promoting proliferation, migration and invasion of lung adenocarcinoma cells [98, 99]. Given the extraordinary variety of transcriptional regulatory machinery discovered in the cell, it is anticipated that more lncRNAs-mediated regulation on hypoxia-induced transcriptional program will be unraveled in the imminent future.

#### HAL-mediated post-transcriptional control

HALs also participate in post-transcriptional regulation including mRNA stability and miRNA-mediated gene silencing.

##### mRNA stability control

Three HALs, *lncRNA-LET* (Long noncoding RNA Low Expression in Tumor), *DANCR* (Differentiation Antagonizing Non-Protein Coding RNA) and *HIF1A-AS2* (HIF1A Antisense RNA 2; also known as *aHIF*), have all been reported to affect *HIF1A* mRNA stability. *lncRNA-LET* expression is generally suppressed in various types of tumors, whereas hypoxia-induced HDAC3 (histone deacetylase 3) could repress its expression by reducing the histone acetylation of the *lncRNA-LET* promoter region [93, 140]. Mechanistically, *lncRNA-LET* is bound to NF90 (nuclear factor 90), which increases NF90 degradation by the proteasome. As RNA binding protein NF90 could stabilize *HIF1A* mRNA [93, 141], the downregulation of *lncRNA-LET* upon hypoxia plays a key role in the stabilization of NF90 protein, thereby increasing *HIF-1A* mRNA stability upon hypoxia and accordingly hypoxia-induced cancer cell invasion [93] (Fig. 1d). Likewise, in nasopharyngeal carcinoma, another oncogenic lncRNA *DANCR* was up-regulated and associated with lymph lode metastasis and poor survival [57]. Through interaction with the NF90/NF45 complex, *DANCR* could increase *HIF1A* mRNA stability, leading to metastasis and disease progression.

In addition, another hypoxia-inducible antisense lncRNA *HIF1A-AS2*, was shown to be up-regulated in various tumors [42, 43, 46, 142, 143] and could differentially regulate HIF-1α and HIF-2α expression during long-term hypoxic conditions [43, 47]. Upon acute hypoxia, HIF-1α and HIF-2α were similarly induced. Interestingly, during prolonged hypoxia, these two proteins were differentially regulated as HIF-1α protein level gradually decreased due to a reduction in its mRNA stability, whereas HIF-2α protein remained upregulated. Meanwhile, long-term hypoxia also induced an increase in *HIF1A-AS2*, whose gene promoter harbors functional HREs. During prolonged hypoxia, *HIF1A-AS2* could bind to its sense counterpart, the *HIF-1A* mRNA 3′-UTR, and possibly expose the AU-rich elements in this region, thereby destabilizing *HIF-1A* mRNA to convey target gene specificity [43, 47]. Paradoxically, *HIF1A-AS2* was also shown to sequester miR153-3p (see next section) to enhance *HIF-1A* expression [44]. Thus, the mode of action of *HIF1A-AS2* is complex and likely context-dependent.

##### miRNA sponges

A wealth of lncRNAs adapt a well-characterized, common mechanism, “ceRNA (competing endogenous RNA)” or “RNA sponges”, to repress miRNA-mediated gene silencing. The ceRNAs compete for shared miRNAs, sequester these miRNAs and diminish their silencing effect on target mRNAs.

Functional manipulations have demonstrated that several HALs, such as *lincRNA-ROR* [87]*, PVT1* [113, 114]*, HIF1A-AS2* [44]*, UCA1* [118]*, HOTAIR* [79]*, FEZF1-AS1* [63]*, ZEB2-AS1* [126] *and H19* [66]*,* could act as a ‘ceRNA’ to reduce individual specific miRNA-mediated *HIF1A* mRNA destabilization and thereby restoring HIF-1α levels and consequently promote cancer progression (Table 1). Specifically, in breast cancer stem cells, by absorbing endogenous miRNA let-7 and aborting let-7-mediated *HIF1A* mRNA suppression, hypoxia-induced H19 could stimulate HIF-1α expression [66] (Fig. 1e). In addition, in glioblastoma, hypoxia-induced H19 up-regulation has been shown to confer an aggressive behavior by sequestering miR-181d and nullifying its suppression on an oncogenic EMT-associated factor, β-catenin [68].

In a similar way, certain HALs could act as a ceRNA to modulate other hypoxia-responsive regulators than HIF-1α. In gastric cancer, *GAPLINC* (Gastric Adenocarcinoma Associated, Positive CD44 Regulator, Long Intergenic Non-Coding RNA) is a HIF-1α direct, transcriptional downstream target, and could promote invasive tumor progression [64]. Mechanistically, *GAPLINC* could serve as a decoy for miR-211-3p to restore the levels of cancer stem cell marker CD44, enhancing tumor progression [65]. Aside from *GAPLINC*, *NORAD* [108]*, UCA1* [119, 120]*, HOTTIP* [82]*, EIF3J-AS1* [59]*, MALAT1* [100]*, FAM201A* [62]*, AGAP2-AS1* [49]*, LINC01436* [84]*, NEAT1* [103]*, NUTF2P3* [109] lncRNAs were shown to function in this way (Table 1). Collectively, in response to hypoxia, the crosstalk among the lncRNA and miRNA transcriptomes build a reciprocal repression feedback network, eliciting concordant shift to transcriptional reprogram. Further exploration of this pertinent co-working group of lncRNAs and miRNAs under hypoxic conditions would help appreciate this emerging additional layer of post-transcriptional regulation governed by HALs.

#### HAL-mediated control of protein activity, stability and/or higher-order complex formation

In addition to acting as ceRNAs to modulate gene expression through interaction with miRNAs, HALs have multiple molecular modes to act at the protein level to further modulate gene expression. One of the hypoxia-induced lncRNAs, *PVT1* (plasmacytoma variant translocation 1), was implicated in cervical cancer progression, likely through its interaction with a multifunctional shuttling protein, nucleolin [115]. In multiple cancer cell lines, HIF-1-induced *lincRNA-p21* provides another example as to how HALs modulate hypoxia response by protein sequestration. Through separate binding to HIF-1α and VHL, *lincRNA-p21* could increase HIF-1α accumulation by disruption of the VHL/HIF-1α interaction and subsequent attenuation of VHL-mediated HIF-1α ubiquitination and degradation [85] (Fig. 1f). Another HIF-1α binding lncRNA *CASC9* (cancer susceptibility candidate 9) is highly expressed in nasopharyngeal carcinoma (NPC) tissues. *CASC9* could interact with and stabilize HIF-1α, promoting the glycolysis and tumorigenesis of NPC cells [53].

Nevertheless, in addition to fine-tuning the activity of one single protein, HALs can also dynamically modulate higher-order protein organizations by serving as scaffolds or molecular decoys. In mesenchymal glioblastoma stem-like cells, through direct binding to two RNA binding proteins, DHX9 (ATP-dependent RNA helicase A) and IGF2BP2 (insulin-like growth factor 2 mRNA-binding protein 2), lncRNA *HIF1A-AS2* could facilitate the interaction between this protein complex and their mRNA target *HMGA1* (high mobility group AT-hook 1), thereby enhancing *HMGA1* expression as well as the downstream molecular response to hypoxic stress [46, 47].

In triple-negative breast cancer (TNBC), *LINK-A* (long intergenic non-coding RNA for kinase activation) has a critical role in the growth factor-induced HIF-1α signaling under normoxic conditions [88]. *LINK-A* is required for the recruitment of BRK (breast tumor kinase) and subsequent enzymatic activation, which is stimulated by HB-EGF (Heparin-binding EGF-like growth factor) signal. HB-EGF mediates the heterodimerization of EGFR (epidermal growth factor receptor) and GPNMB (transmembrane glycoprotein NMB) to form ‘EGFR:GPNMB’ complex. Due to its direct interaction with BRK and LRRK2 (leucine-rich repeat kinase 2), *LINK-A* could recruit these two kinases to EGFR:GPNMB heterodimer, thereby inducing their kinase activities, resulting in HIF-1α phosphorylation: BRK-mediated HIF-1α phosphorylation at Tyr^565^, a phosphorylation preventing the adjacent Pro^564^ hydroxylation of HIF-1α and subsequent HIF-1α degradation under normoxic conditions; and LRRK2-mediated HIF-1α phosphorylation at Ser^797^, which facilitates the interaction of HIF-1α with the transcriptional cofactor p300 [88] (Fig. 1g). In TNBC samples, both *LINK-A* abundance and HIF-1 signaling activation are correlated with cancer progression and shorter survival, revealing potential therapeutic targets for TNBC [88].

An additional novel function of lncRNAs is their structural role in the assembly of nuclear domains. For instance, *MALAT1* (metastasis-associated lung adenocarcinoma transcript 1, also known as *NEAT2*) and *NEAT1* (nuclear enriched abundant transcript 1) are located in two well-characterized nuclear bodies, nuclear speckles and paraspeckles, respectively. Also known as SC35 splicing domains, nuclear speckles are membrane-less compartments and their formation involves “phase-separation” mediated by aggregated lncRNAs and proteins. Being an abundant component of the nuclear speckles, *MALAT1* associates with numerous splicing factors and other SR (serine/arginine-rich) proteins, and is required for their correct localization to the nuclear speckles, although the overall nuclear speckle assembly is not dependent on the abundance of *MALAT1* [144, 145]. So far, the functional involvement of *MALAT1* in RNA splicing in response to hypoxia remains to be determined. In contrast, lncRNA *NEAT1* is shown to be an essential architectural component of nuclear paraspeckles [144, 145]. The precise function of paraspeckles remains largely elusive, but proposed to regulate gene expression via the retention of hyper-edited RNA and other multifunctional factors in the nucleus [104]. Given the functional involvement of both *MALAT1* and *NEAT1* in nuclear structure, further investigation of the extent to which these nuclear structures and their associated transcription reprogramming respond to hypoxia will deepen our understanding of the cellular dynamic response to hypoxia.

#### HAL-mediated control of hypoxia response via unclear mechanism

As listed in Table 1, most of the HALs identified with profound impact on tumorigenesis have not yet been examined in mechanistic detail. However, other reports regarding the same lncRNA with functional characterization might reveal clues about their biological roles in response to hypoxia. For instance, lncRNA *PCGEM1* was found to be overexpressed in gastric cancer, and could be induced by hypoxia [110]. In gastric cancer cells, *PCGEM1* could promote the invasion and metastasis through activating the expression of SNAI1, a key transcription factor of EMT, though the underlying mechanism remains elusive [110]. Notably, in prostate cancer, our group previously reported that the oncogenic *PCGEM1* could promote chromatin recruitment of c-Myc and enhances its transactivation activity through direct physical interaction [146]. As *SNAI1* is a well-characterized downstream gene of c-Myc, the possible functional role of the *PCGEM1*/c-Myc/SNAI1 signaling axis in hypoxia-associated cancer progression warrants further investigation.

In summary, as noted in the above sections, given the relatively large size and the structural flexibility of lncRNAs, it is to be expected that they interact with multiple RNA or protein components and have multi-functions, perhaps in a context-dependent manner. As such, their roles in hypoxia responses and in tumor progression may differ appreciably in different cancer types.

### LncRNAs as predictive biomarkers and therapeutic targets for hypoxic tumor

#### Extracellular vesicles-containing HALs and their biologic effects on tumorigenesis

Extracellular vesicles are effective devices for transporting biomolecules among various cells types [147, 148]. Based on the difference in size and biogenesis, cell-derived extracellular vesicles can be broadly divided into two main categories: exosomes (30–100 nm in diameter) and microvesicles. Together with proteins and other non-coding RNAs, emerging evidence has shown that lncRNAs are packaged into exosomes [149, 150], and the abundance of lncRNAs in exosomes correlates with their expression level in the cell of origin [151]. Through exosomal transfer, several lncRNAs are shown to potentiate cell responses to hypoxia between cancer cells [87], as well as between cancer cell and the associated microenvironment [150]. Table 2 summarizes hypoxia-associated lncRNAs identified extracellularly. For example, *linc-ROR* was found abundant in tumor cells as well as in exosomes derived from tumor cells [87]. It is increased both in cells or exosomes during hypoxia, and it up-regulates HIF-1α expression by absorbing miR-145. By co-culture systems, *linc-ROR*-containing exosomes increase HIF-1a transcription in recipient cells [87]. Hypoxia can shape and fine tune specific macrophage phenotypes in the tumor milieu that are known to promote tumor progression [152]. Chen et al found lncRNA *HISLA* (also known as *LINC01146*), secreted by tumor-associated macrophages, stabilized HIF-1α and enhanced aerobic glycolysis in cancer cells, leading to contagious metabolic reprogramming within tumor regions [150]. *PVT1*, a lncRNA that often co-amplifies with c-myc and functions as miRNA sponge to upregulate HIF-1α expression [153, 154], is another example of exosomal transfer between TAMs (tumor associated macrophages) and cancer cells. *PVT1* is detected in exosomes derived from colon cancer cells, particularly in more aggressive cells [136]. In granulocytic myeloid-derived suppressor cells (G-MDSCs), *PVT1* was up-regulated by HIF-1α under hypoxia and contributed to immunosuppression, given its depletion reduced the suppression of these cells on T-cells and delayed tumor progression [155]. Other exosomal-transferred lncRNAs that are implicated in cancer cells during hypoxia include *UCA1* in bladder cancer for promoting tumor growth and EMT [137], and *CCAT2* for glioma’s resistance to apoptosis and angiogenesis [134].

The functions of lncRNAs in exosomes for tumor progression await to be explored given a significant level of non-coding RNAs are revealed in exosomes (and elevated upon hypoxia) whereas only a small fraction has been studied [149, 150, 156]. Accordingly, it is conceivable that multiple tumor phenotypes and signaling pathways are affected upon exosomal loading. Indeed, by microarray analyses, Mao et al showed hundreds of lncRNAs, together with other transcripts, are changed in endothelial cell recipients of exosomes derived from squamous cancer cells [157]. Importantly, they found exosomes obtained from hypoxic condition facilitate angiogenesis and metastasis better than those obtained from normoxic condition in a xenograft model. Similar effects between normoxic exosomes and hypoxic exosomes on angiogenesis were found in a mouse xenograft model of glioblastoma, with additional effect on accelerating tumor expansion at later stage [158]. The elevation in transcripts by exosomes could result from direct gene transfer, or sequential effects mediated by the transferred genes. By which mechanism lncRNAs are selected to be packaged in the exosomes upon stimuli is not known; nevertheless, these studies revealed exosomes as a means by which hypoxia in the tumor microenvironment facilitates tumor cells to spread and progress.

#### Diagnostic potential of HALs

Several HALs with known oncogenic functions have been detected in patient-derived exosomes, including *H19* in serum from patients with bladder cancer [159], *HOTAIR* in urinary exosomes from patients with urothelial bladder cancer [156], *UCA1* in serum from bladder cancer patients [137], and *HIF1A-AS2* in patients with endometriosis [133]. Future studies aimed at identifying hypoxia-responsive transcripts in extracellular vesicles would surely reveal more players in this aspect. Bearing differential expression patterns between normal and malignant stages and/or tumor size, oncogenic lncRNAs that can be detected extracellularly would potentially serve as non-invasive biomarkers for early detection, prognosis prediction, and disease surveillance. *PCA3*, up-regulated in > 90% of men with prostate cancer, is an example of this [160]. A urine-based assay has been approved by the United States Food and Drug Administration (FDA) since 2012 as an alternative diagnostic test for patients undergoing repeat prostate biopsy or with previous negative prostate biopsy.

As described above, there is considerable evidence indicating hypoxia as a progression factor for tumor development [161]. Hypoxia promotes angiogenesis, tumor metastasis, immune evasion and therapy-resistance. The oxygenation status of tumor was reported to influence local tumor response to radiation treatment, as well as overall survival in a variety of tumors [162–164]. Chemotherapeutic drugs, such as Docetaxel and Sorafenib, also tend to be more effective in normoxic conditions [165, 166]. The hypoxic regions in tumors are infiltrated with cells which promote tumor tolerance (regulatory T-cells, myeloid-derived suppressor cells, and macrophages), while antitumor T-cells are devoid and inhibited by HIF-1α-mediated accumulation of extracellular adenosine [167–169]. PD-L1 (Programmed death-ligand 1), a ligand expressed by tumor cells or myeloid-derived suppressor cells to suppress T-cell’s anti-tumor immunity, is up-regulated by and a direct target of HIF-1α during hypoxia [170]. It has become increasingly apparent that hypoxia in tumors fosters immune suppression and prevents effective immunotherapy. Considering the ill-effects of hypoxia, it is important to detect and to overcome tumor hypoxia even before therapy starts, for the best of patient care.

By far, while there has been a great deal of interest in methodologies to measure hypoxia in patients, an efficient, non-invasive, while sensitive method to detect small regions of hypoxia that frequently occur in the tumors is still lacking [163]. A few metabolic markers (HIF-1α, HIF-2, CA9 and GLUT1) have been used to assess low oxygen tensions by immunohistochemistry [171, 172]; however, the application of them in clinic is limited given that their expressions can be triggered by factors other than hypoxia and that biopsies only represent a small sampling of the tumor. As exosome composition mirrors the hypoxia status of tumors [158], a hypoxia signature may be formulated based on the exosomal hypoxia-responsive transcripts including HALs to evaluate oxygenation in the body for clinical exploitation, once our knowledge is advanced.

#### Therapeutic potential of HALs (targeting hypoxia in cancer therapy, a lncRNA perspective)

Several approaches have been proposed to target hypoxia in tumor [161, 163]. These include drugs that induce cell death selectively in hypoxic cells, e.g. hypoxia-activated prodrugs, or drugs sensitizing hypoxic cells to radiation. Since the adaptive response to hypoxia mainly orients from the transactivation of HIF signaling, some approaches seek to block hypoxia-induced responses by targeting HIFs and the related signaling, or to target pathways that also play pivotal roles in hypoxia adaptation, such as signaling involving mTOR, DNA damage response, and the unfolded protein response. In that regard, HALs that are elevated upon hypoxia and contribute to tumor progression in pre-clinical studies could potentially serve as molecular targets, e.g. *PVT*, *LncHIFCAR*, etc. (see Table 1) [41]. By contrast, HALs that are repressed in order to magnify hypoxia response, such as *lncRNA-LET*, could be induced for therapeutic intervention.

Various strategies have been developed to modulate RNAs. Silencing lncRNAs by small interfering RNAs, antisense oligonucleotides (ASOs), or ribozymes and deoxynucleotides are well demonstrated in pre-clinical studies. Until now, three ASOs and one aptamer therapies have been approved by the FDA for diseases and a handful of others are in clinical trials. The development of short oligonucleotides that fold into three-dimensional structures, aptamers, offers a greater specificity as they target specific structure regions to either mediate RNA degradation or disrupt functional interactions between binding partners [173]. Small molecules that bind to lncRNA and hinder its interaction surface have similar advantages. Additionally, peptide nucleic acids (PNA)-based approach against lncRNAs have been described. *HOTAIR*-targeting PNAs conjugated with pH-low insertion peptide (pHLIP) successfully delivered the anti-lncRNA to the acidic tumor. It blocked the interaction between *HOTAIR* and EZH2, subsequently inhibited *HOTAIR*-EZH2 activity and re-sensitized resistant ovarian tumors to platinum [174].

In any case, an issue all hypoxia-based therapeutics need to consider is the poorly perfused tissue in tumors. In response to their rapid growth, tumor cells secret pro-angiogenic factors such as VEGF to induce vascular formation, yet the constant stimulation leaves tumor vasculature ill-formed and leaky [175]. Simultaneous blockade of HIFs and pro-angiogenic factors has been proposed for targeting tumor hypoxia, in that targeting the angiogenic factors may allow vasculature to mature, resulting more effective blood supply and drug delivery. Another strategy is to relieve oxygen demand by drugs that alleviates oxygen tension in tumors. Papaverine, an FDA-approved drug as a smooth muscle relaxant, was found to inhibit mitochondria complex I and enhance the response to radiotherapy, while well-oxygenated normal tissues were not sensitized [176]. Accordingly, lncRNAs that regulate mitochondria respiration may be considered for targeting tumor hypoxia as an adjuvant treatment.

## Conclusion and future perspectives

Decades of intensive scientific research on hypoxia and HIF biology has greatly contributed to our understanding of oxygen homeostasis. Over the past few years, a substantial increase in our knowledge of the noncoding transcriptomes, while putting on an additional layer of complexity in hypoxia regulation and responses, has advanced our comprehension of hypoxic biology. This review has presented an update of our current insights regarding lncRNAs involved in hypoxia-associated processes, highlighting the diverse mechanisms and functions of hypoxia-associated lncRNAs (HALs). These novel action modes unveil the unanticipated predominance of HALs in the regulation of gene expression under hypoxic conditions and outline the elaborate network among the different types of RNA transcripts, chromatin DNA and protein factors. However, advancement in analytical methodologies and in structural and genomic technologies of RNA are required to open up new important directions for in-depth investigation. For the state-of-the-art methodologies to unveil the functions of lncRNAs, readers are directed to two excellent recent reviews [177, 178], as well as those in this special issue.

The role of HIF in hypoxia responses has been the central topic of most investigations. Indeed, HIF has been shown to be a central regulator of the coding and non-coding transcriptome and tightly associated with cancer risk [12, 179–181]. Most HALs, in particular, are highly responsive to hypoxia and HIF and, in turn, participate in the regulation of the protein-coding genome either *in cis* or *in trans* to offer multiple routes to HIF-mediated gene regulation, implementing both positive and negative feedback loops that either strengthen or repress the hypoxia response. Most notably, the extracellular vesicles-containing HALs could evoke peculiar response to specific cell population, affecting nearby cells and those at a great distance, diversifying the hypoxia response far beyond the previously recognized. The cellular adaptation to hypoxia requires the precisely coordinated regulatory network to cope with the acute, transient and dynamic oxygen deprivation stress in local regions, whereas lncRNAs, with their flexible structure for interaction and quick biogenesis nature, could be uniquely suited to provide rapid, precise and reversible responses to this insult. It is clear that HALs and their downstream targets are shown to confer a series of biological effective responses to hypoxia. Feasibly, this extensive molecular crosstalk between lncRNA and hypoxic signaling cascades may undergo co-evolution to maintain such an exquisite, orchestrated program. Thus, for a comprehensive understanding of hypoxia-associated tumor biology, it is of relevance to characterize the long non-coding transcriptome involved in hypoxia adaptation.

Given the prominent pathological roles of HALs in hypoxia-associated cancer progression, these RNAs could be exploited as useful indicators to define the cancer intrinsic subtypes to aid in precision medicine. Importantly, HALs are often tissue specific and respond to hypoxia in a cell context dependent manner. As such, they are excellent markers for tissue and tumor hypoxia responses. Compared with other bio-molecules, lncRNAs are ideal biomarkers that provide specificity, stability, sensitivity and easy accessibility [38]. Most notably, cell-free lncRNAs or those packed in extracellular vesicles can be detectable in various body fluids [182]. Hence, the genome-wide annotation of tissue-specific HAL signatures could guide development of promising, non-invasive biomarkers for early diagnosis, prognosis and prediction. Although most lncRNA-targeted treatments are still in their infancy stages, the recent success in RNA-based therapeutics holds promises for future technical innovations. With in-depth characterization of the interplay among hypoxia microenvironment and lncRNA function, more HALs could surely accelerate the design of therapeutics for tumor patients, enabling the targeting of the previously undruggable transcriptome in the near future.

## Supplementary information

**Additional file 1: Table S1. **Hypoxia-associated lncRNAs.

## Data Availability

Not Applicable.

## Abbreviations

ASO: Antisense oligonucleotide
bHLH-PAS: Basic helix-loop-helix DNA binding proteins of the PER-ARNT-SIM family
BRK: Breast tumor kinase
ceRNA: competitive endogenous RNA
CRC: Colorectal cancer
CSC: Cancer stem cell
DHX9: ATP-dependent RNA helicase A
EGFR: epidermal growth factor receptor
EMT: Epithelial-mesenchymal transition
FDA: Food and Drug Administration
GC: Gastric cancer
G-MDSCs: Granulocytic myeloid-derived suppressor cells
GPNMB: Transmembrane glycoprotein NMB
GSC: Glioblastoma stem-like cells
HB-EGF: Heparin-binding EGF
HCC: Hepatocellular cancer
HMGA1: High mobility group AT-hook 1
HAL: Hypoxia-associated lncRNAs
HUVECs: Human umbilical vein endothelial cells
IGF2BP2: Insulin-like growth factor 2 mRNA-binding protein 2
LC: Lung cancer
lncRNA: long non-coding RNA
LRRK2: Leucine-rich repeat kinase 2
M-GSCs: Mesenchymal glioblastoma multiforme stem-like cells
ncRNA: non-coding RNA
N.D.: Not determined
NF90: Nuclear factor 90
NSCLC: Non-small cell lung carcinoma
OGT: O-linked N-acetylglucosamine transferase
OS-9: Osteosarcoma amplified-9
OSCC: Oral squamous cell carcinoma
PDAC: Pancreatic ductal adenocarcinoma
PD-L1: Programmed death-ligand 1
pHLIP: pH-low insertion peptide
PNA: Peptide nucleic acids
RCC: Renal Cell Carcinoma
SNCG: Synuclein gamma
TNBC: Triple-negative breast cancer
UTR: Untranslated region
VEGF: Vascular endothelial growth factor
VHL: Von Hippel-Lindau protein
