# Supplementary material for: Signaling in and out: long-noncoding RNAs in tumor hypoxia
Source: J Biomed Sci. 2020 May 5;27:59. doi: 10.1186/s12929-020-00654-x (PMC7201962; doi:10.1186/s12929-020-00654-x)
Supplement: Supplementary file 1 — Additional file 1: Table S1. Hypoxia-associated lncRNAs. [file 12929_2020_654_MOESM1_ESM.docx]

**Table S1 | Hypoxia-associated lncRNAs**

| lncRNA | Synonyms | HGNC ID | Gene/Nucleotide resources |
| --- | --- | --- | --- |
| *AGAP2-AS1* | *AGAP2 Antisense RNA 1, HSALNG0091650, AGAP2-AS1, PUNISHER, LOC100130776* | HGNC:48633 | NR_027032  ENSG00000255737 |
| *BC005927* |  | N.A. | N.A. |
| *CASC9* | *Cancer Susceptibility 9, Esophageal Squamous Cell Carcinoma Associated LncRNA-1, Long Intergenic Non-Protein Coding RNA 981, Esophageal Squamous Cell Carcinoma Sssociated LncRNA-1, ESCC-Associated LncRNA, NONHSAG050548, HSALNG0066261, LINC00981, Linc-JPH1, ESCCAL-1, ESSCAL1* | HGNC: 48906 | NR_103848  ENSG00000249395 |
| *CCAT2* | *Colon Cancer Associated Transcript 2, Non-Coding RNA Involved In Cancer Predisposition 1, Long Intergenic Non-Protein Coding RNA 873, CCAT2, HSALNG0068443, LINC00873, NCCP1* | HGNC: 47044 | NR_109834  ENSG00000280997 |
| *CDKN2B-AS1* | *CDKN2B Antisense RNA 1, CDKN2B-AS1, Prostate Cancer Associated Transcript 12, P15 Antisense RNA, CDKN2B Antisense RNA 1 (Non-Protein Coding), Antisense Noncoding RNA In The INK4 Locus, CDKN2B Antisense RNA (Non-Protein Coding), Antisense RNA In The INK4 Locus, Non-Protein Coding RNA 89, HSALNG0070397, NONHSAG051899, NCRNA00089, CDKN2B-AS, CDKN2BAS, PCAT12, ANRIL, P15AS* | HGNC: 34341 | NR_003529  ENSG00000240498 |
| *CPS1-IT1* | *CPS1 Intronic Transcript 1, CPS1 Intronic Transcript (Non-Protein Coding), NONHSAG030410, HSALNG0021972, CPS1-IT, CPS1IT1, PRO0132, CPS1IT* | HGNC: 30102 | NR_002763  ENSG00000280837 |
| *DANCR* | *Differentiation Antagonizing Non-Protein Coding RNA, KIAA0114, Small Nucleolar RNA Host Gene 13 (Non-Protein Coding), Adipogenesis Up-Regulated Transcript 2, Anti-Differentiation Noncoding RNA, Anti-Differentiation ncRNA, SNHG13, ANCR, Anti Differentiation ncRNA Protein, NONHSAG037936, HSALNG0034419, LncRNA-ANCR, AGU2* | HGNC: 28964 | NR_024031  ENSG00000226950 |
| *DARS-AS1* | *DARS Antisense RNA 1, HSALNG0019085, NONHSAG029377* | HGNC: 40170 | NR_110199  ENSG00000231890 |
| *EIF3J-DT* | *EIF3J Divergent Transcript, EIF3J Antisense RNA 1 (Head To Head), EIF3J-DT, NONHSAG016741, HSALNG0105564, EIF3J-AS1* | HGNC: 48616 | NR_034170  ENSG00000179523 |
| *FALEC* | *Focally Amplified Long Non-Coding RNA In Epithelial Cancer, Long Intergenic Non-Protein Coding RNA 568, Focally Amplified LncRNA On Chromosome 1, FALEC, Noncoding RNA-Activating 1, NONHSAG002854, HSALNG0007045, LINC00568, NcRNA-A1, FAL1* | HGNC: 43713 | NR_051960  ENSG00000228126 |
| *FAM201A* | *Family With Sequence Similarity 201 Member A, C9orf122, Family With Sequence Similarity 201 Member A, Chromosome 9 Open Reading Frame 122, Protein FAM201A, NONHSAG052204, HSALNG0071146* | HGNC: 27239 | NR_027294  ENSG00000204860 |
| *FEZF1-AS1* | *FEZF1 Antisense RNA 1, NONHSAG048666, HSALNG0060982* | HGNC: 41001 | NR_036484  ENSG00000230316 |
| *GAPLINC* | *Gastric Adenocarcinoma Associated, Positive CD44 Regulator, Long Intergenic Non-Coding RNA, Gastric Adenocarcinoma Predictive Long Intergenic Noncoding RNA, Long Intergenic Non-Protein Coding RNA 1540, LncRNA-Uc002kmd.1, TCONS_00026238, HSALNG0119710, NONHSAG023235, RP11-838N2.4, LINC01540* | HGNC: 51308 | NR_110429  ENSG00000266835 |
| *H19* | *H19 Imprinted Maternally Expressed Transcript, Imprinted Maternally Expressed Transcript (Non-Protein Coding), Imprinted Maternally Expressed Untranslated mRNA, Long Intergenic Non-Protein Coding RNA 8, Adult Skeletal Muscle, Non-Protein Coding RNA 8, MIR675 Host Gene, HSALNG0082178, NONHSAG007409, MIR675 Host, NCRNA00008, LINC00008, D11S813E, MIR675HG, ASM1, ASM, BWS, WT2* | HGNC: 4713 | NR_002196  ENSG00000130600 |
| *HAS2-AS1* | *HAS2 Antisense RNA 1, Hyaluronan Synthase 2 Antisense, HAS2 Antisense RNA (Non-Protein Coding), Non-Protein Coding RNA 77, HSALNG0068110, NONHSAG051144, NONHSAG051141, HSALNG0068112, NCRNA00077, Lnc-ZHX2-6, HAS2-AS, HAS2AS, HASNT* | HGNC: 34340 | NR_002835  ENSG00000248690 |
| *HIF1A-AS2* | *HIF1A antisense RNA 2, aHIF, 3'aHIF-1A, HSALNG0101780, Lnc-TMEM30B-9, NONHSAG015182, NONHSAG015184* | HGNC: 43015 | NR_045406  ENSG00000258667 |
| *HIF2PUT* | *TCONS_00004241* | N.A. | TCONS_00004241 |
| *HOTAIR* | *HOX Transcript Antisense RNA, HOX Transcript Antisense RNA (Non-Protein Coding), HOXC Cluster Antisense RNA 4 (Non-Protein Coding), Hox Transcript Antisense Intergenic RNA, Non-Protein Coding RNA 72, NONHSAG011264, HSALNG0091318, HOXC11-AS1, NCRNA00072, HOXC-AS4, HOXAS* | HGNC: 33510 | NR_003716  ENSG00000228630 |
| *HOTTIP* | *HOXA Distal Transcript Antisense RNA, HOXA Cluster Antisense RNA 6 (Non-Protein Coding), HOXA13 Antisense RNA 1 (Non-Protein Coding), HoxA Transcript At The Distal Tip, HOXA Transcript At The Distal Tip, Non-Protein Coding RNA 213, NONHSAG047197, HSALNG0056871, HOXA13-AS1, NCRNA00213, HOXA-AS6* | HGNC: 37461 | NR_037843  ENSG00000243766 |
| *IDH1-AS1* | *IDH1 Antisense RNA 1, HSALNG0021909, NONHSAG030384* | HGNC: 40292 | INSDC AI492612  ENSG00000231908 |
| *LINC01139* | *Long Intergenic Non-Protein Coding RNA 1139, Long Intergenic Non-Coding RNA For Kinase Activation, LOC339535, LINC01139, TCONS_00000027, HSALNG0011845, NONHSAG004770, LINK-A, LINKA* | HGNC: 27924 | NR_015407  ENSG00000215808 |
| *LINC01146* | *Long Intergenic Non-Protein Coding RNA 1146, LINC01146, HSALNG0103023, NONHSAG015652, NONHSAG015653, HIF-1α-stabilizing long noncoding RNA, HISLA* | HGNC: 49467 | NR_046094  ENSG00000258867 |
| *LINC01436* | *Long Intergenic Non-Protein Coding RNA 1436, LINC01436, NONHSAG032761, HSALNG0132900* | HGNC:50754 | NR_110419  ENSG00000231106 |
| *linc-ROR* | *Long Intergenic Non-Protein Coding RNA, Regulator Of Reprogramming, LincRNA-RoR, LINC-ROR, LincRNA-ST8SIA3, HSALNG0121746, NONHSAG023930, ROR* | HGNC: 43773 | NR_048536  ENSG00000258609 |
| *lnc-METTL16-2* | *lncRNA-HAL, ENST00000575402.1, RP1-59D14.6-001, OTTHUMT00000438874.1, NONHSAT144953* | N.A. | ENSG00000261903.1 |
| *lnc-NDRG1-1* | *HSALNG0068730, NONHSAG051331, Lnc-NDRG1-1, NDRG-OT1* | N.A. | N.A. |
| *lnc-P2RY1-1* | *lncRNA-SARCC, ENST00000460407, Lnc-P2RY1-1, ENST00000460407.1, RP11-38P22.2-001, OTTHUMT00000356944.1, NONHSAT092772, ENSG00000241732, RP11-38P22.2, ENSG00000241732.1, OTTHUMG00000159695.1, NONHSAG036423* | N.A. | ENST00000460407 |
| *lncRNA-AK058003* |  | N.A. | AK058003.1 |
| *lncRNA-CF129145.1* | *CF129* | N.A. | lncRNA-CF129145.1 |
| *lncRNA-EFNA3* |  | N.A. | N.A. |
| *MALAT1* | *Metastasis Associated Lung Adenocarcinoma Transcript 1, Nuclear Paraspeckle Assembly Transcript 2 (Non-Protein Coding), Long Intergenic Non-Protein Coding RNA 47, Nuclear Enriched Abundant Transcript 2, Hepcarcin, Metastasis Associated In Lung Adenocarcinoma Transcript 1, Non-Protein Coding RNA 47, HSALNG0084905, NONHSAG008675, NCRNA00047, LINC00047, PRO2853, NEAT2, HCN, PRO1073, MALAT-1* | HGNC: 29665 | NR_002819  ENSG00000251562 |
| *MEG3* | *Maternally Expressed 3, Long Intergenic Non-Protein Coding RNA 23, Very Putative Protein From MEG3 Locus, Non-Protein Coding RNA 23, Onco-LncRNA-83, NONHSAG015923, HSALNG0103778, Lnc-DLK1-35, NCRNA00023, LINC00023, PRO0518, PRO2160, Prebp1, FP504, GTL2* | HGNC: 14575 | NR_002766  ENSG00000214548 |
| *MIR31HG* | *MIR31 host gene, long noncoding HIF-1α co-activating RNA, LncHIFCAR, hsa-lnc-31, LOC554202* | HGNC: 37187 | NR_027054  ENSG00000171889 |
| *MTA2TR* | *MTA2 transcriptional regulator RNA* | N.A. | AF083120.1 |
| *NEAT1* | *Nuclear Paraspeckle Assembly Transcript 1, Nuclear Paraspeckle Assembly Transcript 1 (Non-Protein Coding), Long Intergenic Non-Protein Coding RNA 84, Nuclear Enriched Abundant Transcript 1, Virus Inducible Non-Coding RNA, Trophoblast Derived Non-Protein Coding RNA, Trophoblast MHC Class II Suppressor, Trophoblast-Derived Noncoding RNA, Non-Protein Coding RNA 84, MENepsilon/Beta, HSALNG0084892, NONHSAG008670, NCRNA00084, LINC00084, TncRNA, VINC* | HGNC: 30815 | NR_028272  ENSG00000245532 |
| *NORAD* | *Non-Coding RNA Activated By DNA Damage, Long Intergenic Non-Protein Coding RNA 657, NONHSAG031696, HSALNG0129818, LINC00657* | HGNC: 44311 | NR_027451  ENSG00000260032 |
| *NPTN-IT1* | *NPTN intronic transcript 1,* *lncRNA-LET, HSALNG0107117, NONHSAG017379, “long non-coding RNA, low expression in tumor”* | HGNC: 45091 | NR_103844  ENSG00000281183 |
| *NUTF2P3* | *Nuclear Transport Factor 2 Pseudogene, NUTF2P3-001* | HGNC:50452 | NG_044239  ENSG00000228248 |
| *PCGEM1* | *PCGEM1 Prostate-Specific Transcript, Prostate-Specific Transcript (Non-Protein Coding), Prostate-Specific Transcript 1 (Non-Protein Coding), Long Intergenic Non-Protein Coding RNA 71, Prostate Cancer Gene Expression Marker 1, Prostate Cancer Associated Transcript 9, Non-Protein Coding RNA 71, HSALNG0021208, NONHSAG030128, NONHSAG030129, NCRNA00071, LINC00071, Pcgem1, PCAT9* | HGNC: 30145 | NR_002769  ENSG00000227418 |
| *PVT1* | *Pvt1 Oncogene, Pvt-1 (Murine) Oncogene Homolog, MYC Activator, Long Intergenic Non-Protein Coding RNA 79, Pvt1 Oncogene (Non-Protein Coding), “MIR1204, MIR1205, MIR1206 And MIR1207 Host Gene”, “MIR1204, MIR1205, MIR1206 And MIR1207 Host”, Plasmacytoma Variant Translocation 1, Oncogene PVT-1 (MYC Activator), Pvt1 Oncogene Homolog (Mouse), Non-Protein Coding RNA 79, Onco-LncRNA-100, HSALNG0068477, NONHSAG051258, NCRNA00079, LINC00079, MIR1204HG* | HGNC: 9709 | NR_003367  ENSG00000249859 |
| *RAB4B-EGLN2* | *RERT-lncRNA, RAB4B-EGLN2 Readthrough (NMD Candidate), RAB4B-EGLN2 Readthrough Long Non-Coding RNA, Prolyl Hydroxylase Domain-Containing Protein 1, Hypoxia-Inducible Factor Prolyl Hydroxylase 1, RAB4B-EGLN2 Readthrough (Non-Protein Coding), HCG2044074, Isoform CRA_b, HCG1995540, Isoform CRA_c, HIF-Prolyl Hydroxylase 1, Estrogen-Induced Tag 6, Egl Nine Homolog 2, HIF-PH1, EGLN2, EIT-6, HPH-1, HPH-3, EIT6, PHD1* | HGNC: 44465 | NR_037791  ENSG00000171570 |
| *RP11-225B17.2* | *ccRCC prognosis-associated transcript 4, CRPAT4, OTTHUMG00000186213* | N.A. | ENSG00000273014.1 |
| *RPL13AP23* | *Ribosomal protein L13a pseudogene 23, RPL13A_10_1240, RP 11–571M6.1, ENST00000480739* | HGNC: 36498 | ENSG00000242990 |
| *TP53COR1* | *Tumor protein p53 pathway corepressor 1, linc-p21, lincRNA-p21, Trp53cor1, Ak144811, Gm16197* | HGNC:43652 | NCBI Gene: 102800311  INSDC CD515754 |
| *UBE2CP3* | *Ubiquitin Conjugating Enzyme E2 C Pseudogene 3, Ubiquitin Conjugating Enzyme E2C Pseudogene 3, Ubiquitin-Conjugating Enzyme E2C Pseudogene 3* | HGNC: 43552 | NG_022057  ENSG00000250384 |
| *UCA1* | *Urothelial Cancer Associated 1, Long Intergenic Non-Protein Coding RNA 178, Cancer Up-Regulated Drug Resistant, Onco-LncRNA-36, HSALNG0124349, NONHSAG025011, HSALNG0124398, UCA1 Protein, NCRNA00178, LINC00178, UCAT1, CUDR* | HGNC: 37126 | NR_015379  ENSG00000214049 |
| *WSPAR* | *WNT Signaling Pathway Activating Non-Coding RNA, TCONS_00009511, HSALNG0044933, NONHSAG041563, LncTCF7* | HGNC:51639 | NR_131252  ENSG00000249073 |
| *WT1-AS* | *WT1 Antisense RNA, Wilms Tumor Upstream Neighbor 1, Wilms Tumor Associated Protein, WT1-AS, WIT-1, WIT1, Putative Wilms Tumor Upstream Neighbor 1 Gene Protein, Wilms Tumor-Associated Antisense RNA, NONHSAG007984, HSALNG0083476, WT1-AS1, WT1AS* | HGNC: 18135 | NR_023920  ENSG00000183242 |
| *ZEB2-AS1* | *ZEB2 Antisense RNA 1, ZEB2 Natural Antisense Transcript, ZEB2-AS1, ZEB2 Antisense RNA (Non-Protein Coding), HSALNG0019362, NONHSAG029467, ZEB2NAT, ZEB2-AS, ZEB2AS* | HGNC: 37149 | NR_040248  ENSG00000238057 |
| *ZEBTR* | *ZEB1 Transcriptional Regulator RNA, LncRNA-BX111887, LncRNA-BX111, BX111* | HGNC: 53967 | NCBI Gene 113391337  INSDC BX111887 |

All the information is mainly adapted from GeneCards (genecards.org) and HGNC (HUGO Gene nomenclature Committee; genenames.org). Abbreviation: *N.A.* Not available.
